# Developing and validating a nomogram for predicting endoscopic hemostasis failure in cirrhotic patients with esophageal variceal bleeding

**DOI:** 10.3389/fmed.2025.1670759

**Published:** 2025-10-07

**Authors:** Yali Guo, Hui Ouyang, Jingling Su, Mingrong Zhong, Wenzhong Huang, Mingcheng Huang, Chenxi Xie

**Affiliations:** ^1^Endoscopy Center, Xiamen Hospital of Traditional Chinese Medicine (Xiamen Hospital Affiliated with Dongzhimen Hospital of Beijing University of Chinese Medicine), Xiamen, China; ^2^Department of Digestive Medicine Center, The Seventh Affiliated Hospital, Sun Yat-sen University, Shenzhen, China; ^3^Department of Gastroenterology, Clinical Research Center for Gut Microbiota and Digestive Diseases of Fujian Province, The National Key Clinical Specialty, Zhongshan Hospital Xiamen University, School of Medicine, Xiamen University, Xiamen, China; ^4^Department of Nephrology, Center of Kidney and Urology, The Seventh Affiliated Hospital, Sun Yat-sen University, Shenzhen, China; ^5^Xiamen Key Laboratory of Intestinal Microbiome and Human Health, Zhongshan Hospital Xiamen University, Xiamen, China; ^6^Department of Digestive Disease, Institute for Microbial Ecology, School of Medicine, Xiamen University, Xiamen, China

**Keywords:** liver cirrhosis, esophagogastric variceal bleeding, endoscopic treatment, nomogram, endoscopic hemostasis failure

## Abstract

**Background and aims:**

This study aimed to create and validate a model to predict the failure of endoscopic hemostasis in Chinese cirrhosis patients with acute esophagogastric variceal bleeding (EGVB), enabling early identification of high-risk individuals.

**Methods:**

A retrospective study analyzed 296 cirrhotic patients with EGVB who received emergency endoscopic therapy from January 2020 to February 2025. Patients were divided into success (*n* = 273) and failure (*n* = 23, defined as bleeding recurrence within 5 days) groups. LASSO regression optimized variable selection, and multivariate logistic regression identified independent predictors to create a nomogram. Internal validation used Bootstrap resampling (500 iterations). Model performance was assessed using ROC curves, calibration plots, and decision curve analysis (DCA), and compared with CTP (Child-Turcotte-Pugh), MELD (Model for End-Stage Liver Disease), and Rockall scores.

**Results:**

The cumulative incidence of endoscopic failure was observed to be 7.8%. Independent predictors identified included a shock index (SI) > 1.2 (OR = 5.447), the presence of a red color (RC) sign (OR = 10.005), active bleeding observed during endoscopy (OR = 5.962), and the CTP (OR = 1.584). The nomogram exhibited superior discriminatory power with an AUC of 0.890 (95% CI: 0.820–0.960), outperforming the CTP (AUC = 0.771, 95% CI: 0.656–0.886; *P* < 0.001), MELD (AUC = 0.733, 95% CI: 0.616–0.849; *P* < 0.001), and Rockall (AUC = 0.656, 95% CI: 0.545–0.768; *P* < 0.001). Calibration was satisfactory as indicated by the Hosmer–Lemeshow test (χ^2^ = 10.021, *P* = 0.263). DCA demonstrated a clinical net benefit across a broad range of thresholds.

**Conclusion:**

A validated nomogram that integrates the SI, RC sign, active bleeding, and CTP provides an effective prediction of the risk of endoscopic hemostasis failure in patients with cirrhotic EGVB, thereby facilitating timely intervention.

## 1 Introduction

Esophagogastric variceal bleeding (EGVB) represents the most critical complication associated with portal hypertension, exhibiting an acute-phase mortality rate of 20%−30% (1). Both national and international guidelines advocate for endoscopic variceal hemostasis as a highly effective and essential treatment modality for cirrhotic EGVB (1–3). Despite these recommendations, clinical practice reveals that 15%−25% of patients experience hemostasis failure or early rebleeding (4), resulting in a significant increase in mortality to over 35% within 48 h (5). In instances where endoscopic hemostasis is unsuccessful, urgent interventional surgery or secondary endoscopic intervention becomes necessary, thereby escalating the healthcare burden and complicating treatment. Consequently, the early identification of patients at high risk for endoscopic treatment failure and the formulation of individualized treatment plans have emerged as crucial steps in enhancing patient prognosis. Currently, widely utilized prognostic assessment tools for cirrhosis, such as the Child-Turcotte-Pugh (CTP) classification and the Model for End-Stage Liver Disease (MELD), while capable of reflecting liver function reserve and overall mortality risk, predominantly rely on static laboratory indicators. These tools often fail to incorporate dynamic endoscopic characteristics and hemodynamic parameters. A retrospective study indicated that the CTP and MELD scores exhibit limited predictive efficacy for endoscopic treatment failure in patients with EGVB, with both scores demonstrating an AUC of < 0.7 (6). This limitation may be attributed to their disregard for local lesion characteristics. Furthermore, the Rockall score, a commonly employed assessment tool for upper gastrointestinal bleeding, has shown a significant reduction in predictive specificity within the context of EGVB, achieving an AUC of only 0.67 (7), likely due to the confounding influence of non-variceal bleeding factors.

In light of the aforementioned limitations, this study endeavors to develop and validate a visual nomogram model utilizing multidimensional clinical data to accurately predict the risk of endoscopic treatment failure in patients with cirrhotic EGVB. By incorporating critical variables, including demographic characteristics, laboratory indicators, imaging parameters, and endoscopic findings, the study seeks to identify high-risk individuals. This identification aims to assist clinicians in proactively preparing rescue measures, such as three-lumen balloon tamponade and emergency transjugular intrahepatic portosystemic shunt (TIPS), as well as to enhance postoperative monitoring and intervention strategies, thereby effectively reducing the incidence of complications associated with treatment failure.

## 2 Materials and methods

### 2.1 Study subjects

This investigation is a single-center, retrospective study conducted at the Dongzhimen Hospital Xiamen Hospital of Beijing University of Chinese Medicine (Xiamen Traditional Chinese Medicine Hospital). It involved the consecutive enrollment of patients diagnosed with cirrhotic EGVB who underwent endoscopic hemostasis between January 2020 and February 2025. The inclusion criteria were as follows: (1) patients meeting the accepted diagnostic criteria for liver cirrhosis (8); (2) patients undergoing endoscopic treatment for EGVB for the first time; (3) individuals who provided informed consent prior to the procedure and received standard endoscopic hemostasis treatment, including band ligation, tissue adhesive injection, or sclerotherapy; (4) all procedures were performed by experienced endoscopists with a minimum of five years of experience in the department and an annual procedural volume of no < 50 related interventions. The exclusion criteria were: (1) bleeding attributed to non-cirrhotic EGVB causes, such as peptic ulcers, Mallory-Weiss tears, upper gastrointestinal malignancies, or vascular malformations, as identified during endoscopy or by subsequent investigations; (2) cases where endoscopic examination failed to clearly identify the source of bleeding. The Ethics Committee of Dongzhimen Hospital Xiamen Hospital of Beijing University of Chinese Medicine (Xiamen Traditional Chinese Medicine Hospital) granted approval for this study (Ethics Review No. 2024-K033-01). The study adhered to the ethical principles outlined in the 2013 Declaration of Helsinki. Given the retrospective nature of the study, the requirement for written informed consent was waived.

### 2.2 Timing and methods of treatment

(1) Timing of treatment: all patients presenting with EGVB underwent gastroscopy within 24 h of hospital admission. For those experiencing refractory hemorrhagic shock, endoscopic intervention was conducted at the earliest opportunity under general anesthesia with tracheal intubation and intensive care unit (ICU) support, following comprehensive understanding and informed consent from family members. In this study, upon admission, all patients were immediately administered fasting protocols, fluid resuscitation, vasoactive agents, and prophylactic antibiotics. If the patient's hemodynamic status stabilized following these interventions, EGVB treatment was performed in the operating room under tracheal intubation within 24 h. Conversely, if hemodynamic instability persisted, EGVB was conducted immediately at the ICU bedside under tracheal intubation.
(2) Treatment methods: all patients underwent emergency endoscopic interventions guided by the LDRF classification. The procedures included Endoscopic Variceal Ligation (EVL), Endoscopic Injection Sclerotherapy (EIS), Endoscopic Tissue Adhesive (ETA), or a combination of sclerosant and tissue glue injection. In cases where emergency endoscopic treatment was unsuccessful, alternative measures such as tamponade using a three-lumen balloon catheter, repeated endoscopic interventions, or emergency TIPS were implemented. All emergency endoscopic procedures were conducted by physicians with a minimum of 5 years of experience, in accordance with the “Guidelines for the Prevention and Treatment of Esophageal and Gastric Variceal Hemorrhage due to Portal Hypertension in Cirrhosis” (8).

### 2.3 Evaluation indicators

(1) Failure of emergency endoscopic hemostasis: in accordance with the Baveno VII consensus (2), failure of emergency endoscopic hemostasis is characterized by the inability to achieve bleeding control or the occurrence of rebleeding within a five-day period.
(2) Diagnosis of failure of emergency endoscopic treatment and rebleeding: this diagnosis encompasses the recurrence of EGVB, which may manifest as hematemesis, melena, or hematochezia. Additional diagnostic criteria include a reduction in systolic blood pressure exceeding 20 mmHg, an increase in heart rate exceeding 20 beats per minute, or a decrease in hemoglobin levels exceeding 30 g/L in the absence of blood transfusion (1).
(3) Shock index (SI): the SI is calculated as the ratio of heart rate (beats per minute) to systolic blood pressure (mmHg) (9).
(4) Bleeding activity: defined as the direct endoscopic visualization of oozing, spurting, or actively adherent blood clots on esophageal or gastric varices, or the detection of fresh blood in the absence of these signs, following the exclusion of non-variceal sources.

### 2.4 Clinical variables

Data were meticulously collected and retrieved from the electronic medical record system, encompassing demographic information, laboratory indicators, imaging parameters, and endoscopic features at the time of patient admission. The variables included age, gender, etiology, presence of ascites, liver cancer, timing of endoscopic treatment, diameter of varices as observed via gastroscopy, Sarin classification, presence of red color signs, active bleeding, treatment methods, as well as heart rate and blood pressure upon admission. Additionally, the initial venous blood laboratory test conducted post-admission was recorded. Furthermore, the SI, CTP score and classification, Model for MELD score, and Rockall score were computed. All data were independently collected and verified by two researchers to ensure accuracy. Drawing on evidence from a single-center retrospective study involving 58,336 emergency patients by Balhara et al. (10), which demonstrated a significant association between an initial SI > 1.2 and in-hospital mortality, this study employed an SI cutoff value of 1.2 to categorize patients into two groups (SI ≤ 1.2 vs. SI > 1.2).

### 2.5 Statistical methods

Continuous variables with a normal distribution were reported as mean ± standard deviation and analyzed using a two-tailed Student's *t*-test. Non-normally distributed variables were presented as median and interquartile range (IQR) and evaluated with the Mann–Whitney *U* test. Categorical data were shown as frequencies and percentages and assessed with the Chi-square or Fisher's exact test. LASSO regression was used for variable selection. Predictive models were developed via multivariable logistic regression, and nomograms were created using the rms package in R. Model performance was assessed with ROC curves, calibration plots, and DCA, with internal validation via bootstrap resampling (500 iterations). Statistical analyses were conducted in R version 4.0.3, with significance at a two-sided *P* value < 0.05.

## 3 Results

### 3.1 Comparison of patient characteristics

**Figure 1:**
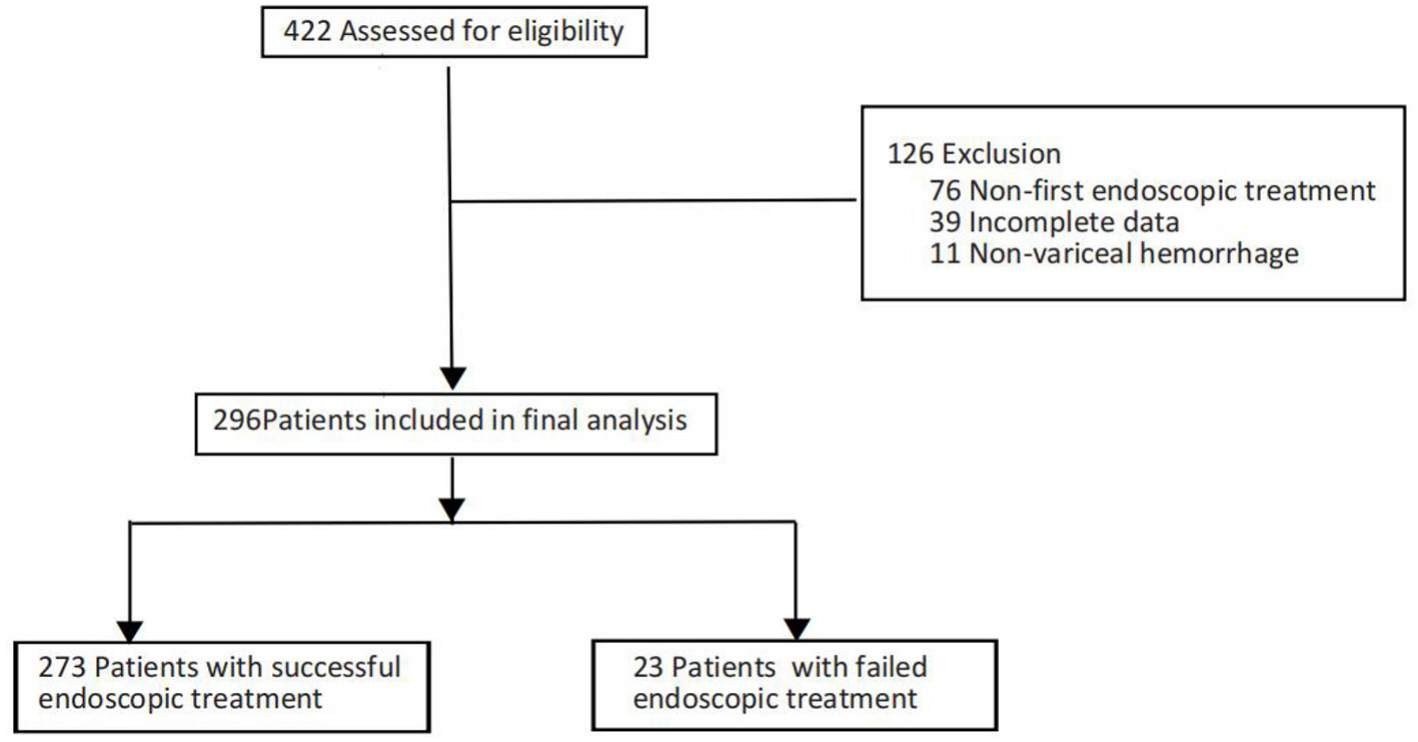
Flowchart of the process of patient enrollment.

Between January 1, 2020, and February 1, 2025, a total of 422 patients presenting with cirrhotic EGVB received endoscopic hemostatic intervention at the endoscopy center of our hospital. Following the application of exclusion criteria, 126 patients were omitted from the study. Consequently, 296 patients remained for further analysis. The rate of endoscopic hemostasis failure was determined to be 7.8%. The process of patient selection is illustrated in Figure 1, while comprehensive demographic and clinical data are provided in Table 1.

**Table 1:** Comparison of general data between the successful group and the failed group.

**Patient characteristics**	**All (*n* = 296)**	**Treatment success group (*n* = 273)**	**Treatment failure group (*n* = 23)**	***P*-value**
Male, sex	224 (75.68)	206 (75.46)	18 (78.26)	0.962
Age, year	54.00 [49.00; 61.25]	54.00 [49.00; 62.00]	51.00 [47.00; 57.50]	0.096
Shock Index >1.2	58 (19.59)	43 (15.75)	15 (65.22)	< 0.001^***^
Hepatic Carcinoma	42 (14.19)	36 (13.19)	6 (26.09)	0.114
RC sign	211 (71.2)	190 (69.60)	21 (91.30)	0.049^*^
Active bleeding	64 (21.62)	57 (20.88)	7 (30.43)	0.295
EV diameter ≥ 1cm	86 (29.05)	78 (28.57)	8 (34.78)	0.696
Rockall score	4.00 [3.00; 5.00]	4.00 [3.00; 5.00]	5.00 [4.00; 6.50]	0.010^*^
MELD score	11.42 [9.21; 14.47]	11.17 [9.14; 14.11]	15.24 [11.78; 20.48]	< 0.001^***^
CTP score	8.00 [7.00; 9.00]	8.00 [7.00; 9.00]	11.00 [8.00; 13.00]	< 0.001^***^
**CTP grade**				
A	62 (20.95)	60 (21.98)	2 (8.70)	< 0.001^***^
B	165 (55.74)	158 (57.88)	7 (30.43)	
C	69 (23.31)	55 (20.15)	14 (60.87)	
**Etiology**				
Viral	192 (64.86)	180 (65.93)	12 (52.17)	0.118
Alcoholic	60 (20.27)	56 (20.51)	4 (17.39)	
Others	44 (14.86)	37 (13.55)	7 (30.43)	
**Sarin classification**				
GOV1	227 (76.69)	209 (76.56)	18 (78.26)	1.000
GOV2	65 (21.96)	60 (21.98)	5 (21.74)	
IGV1	4 (1.35)	4 (1.47)	0 (0.00)	
**Ascites**				
None	65 (21.96)	60 (21.98)	5 (21.74)	0.911
Grade 1	177 (59.80)	164 (60.07)	13 (56.52)	
Grade 2–3	54 (18.24)	49 (17.95)	5 (21.74)	
**Hemostasis**				
EVL/EVL+ETA	200 (67.57)	184 (67.40)	16 (69.57)	1.000
EIS/EIS+ETA	29 (9.80)	27 (9.89)	2 (8.70)	
ETA	67 (22.64)	62 (22.71)	5 (21.74)	
**Management timing**				
≤ 12 h	130 (43.92)	112 (41.03)	18 (78.26)	0.001^**^
>12 h	166 (56.08)	161 (58.97)	5 (21.74)	
**Laboratory data**				
WBC, × 10^9^/L	4.90 [3.30; 7.20]	4.80 [3.30; 6.90]	7.80 [5.20; 15.20]	< 0.001^***^
NE, %	72.90 [65.60; 81.15]	72.00 [64.80; 80.70]	79.60 [76.95; 85.20]	0.001 ^**^
HGB, g/L	75.00 [64.00; 90.25]	77.00 [64.00; 93.00]	67.00 [55.50; 72.50]	0.002^**^
PLT, × 10^9^/L	75.00 [54.00; 106.50]	74.00 [54.00; 104.00]	99.00 [61.00; 117.00]	0.191
PT, s	15.20 [13.90; 17.42]	15.10 [13.90; 17.10]	20.20 [15.15; 23.10]	0.001^**^
PT prolongation, s	2.10 [0.80; 4.32]	2.00 [0.80; 4.00]	7.10 [2.05; 10.00]	0.001^**^
INR	1.34 [1.22; 1.55]	1.34 [1.22; 1.52]	1.79 [1.33; 2.03]	0.001^**^
APTT, s	32.00 [29.20; 35.52]	32.00 [29.20; 35.30]	34.20 [29.60; 48.05]	0.142
ALB, g/L	30.00 [27.00; 34.00]	30.00 [28.00; 34.00]	29.00 [24.00; 33.00]	0.055
TBIL, umol/L	26.00 [18.55; 38.42]	25.00 [18.20; 37.00]	41.00 [22.00; 99.35]	0.003^**^
ALT, U/L	25.50 [18.00; 40.00]	25.00 [18.00; 40.00]	26.00 [14.50; 80.50]	0.442
AST, U/L	38.00 [27.00; 60.00]	38.00 [27.00; 57.00]	36.00 [27.50; 239.50]	0.155
Cr, umol/L	66.00 [55.00; 77.00]	65.80 [55.00; 74.60]	78.00 [70.50; 95.50]	0.001^**^
Na, umol/L	139.00 [138.00; 141.00]	139.00 [138.00; 141.00]	139.00 [136.50; 140.00]	0.486

Data are presented as number (%) or median (interquartile range).

^*^P < 0.05, ^**^P < 0.01, ^***^P < 0.001.

### 3.2 Risk factors for endoscopic treatment failure

Among the cohort of 296 patients, endoscopic hemostasis was successfully achieved in 273 individuals, while it failed in 23. The median age did not differ significantly between these two groups. The predominant etiology of cirrhotic EGVB was viral, accounting for 64.86% (192/296) of cases, followed by alcoholic causes at 20.27% (60/296), and other causes at 14.86% (44/296). No significant differences were observed between the groups regarding gender, age, etiology, presence of hepatocellular carcinoma (HCC), ascites grade, endoscopic active bleeding, variceal diameter, Sarin classification, or treatment modality (*P* > 0.05). However, patients who experienced treatment failure exhibited a significantly higher incidence of a shock index, the presence of red color signs on endoscopy, and a shorter interval from admission to the initiation of endoscopic treatment compared to those with successful outcomes. Upon admission, patients with treatment failure demonstrated significantly elevated levels of serum white blood cell count (WBC), neutrophil percentage (NE%), hemoglobin (HGB), prothrombin time (PT), PT prolongation, international normalized ratio (INR), total bilirubin (TBIL), and creatinine (Cr) compared to those who were successfully treated. Among the various scoring systems, the CTP classification, CTP score, MELD score, and Rockall score demonstrated significant differences between treatment success and treatment failure (Table 1).

**Figure 2:**
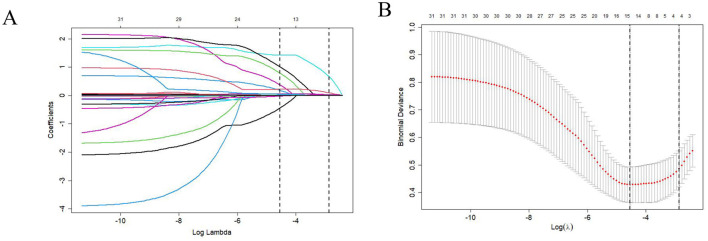
**(A)** Feature selection was performed using a LASSO binary logistic regression model, and coefficient profiles were plotted for all 31 coefficients (including the intercept) to illustrate their trends with respect to the log(lambda) sequence. **(B)** Parameter selection in the LASSO model used tenfold cross-validation via minimum criterion: partial likelihood deviation (binomial deviation) curves and logarithmic (lambda) curves were plotted. Use the minimum standard and 1 se (1-SE standard) of the minimum standard to draw a vertical dashed line at the optimal value. The optimal lambda produced four nonzero coefficients. LASSO, least absolute shrinkage and selection operator.

Feature selection was performed using LASSO regression with 10-fold cross-validation on 30 clinical variables. To prioritize predictive accuracy and minimize the risk of excluding potentially important factors, the lambda value yielding the minimum cross-validation error (lambda_min) was selected. This process resulted in the identification of 15 non-zero coefficients (Figure 2). The selected features included time to endoscopic treatment, age, etiology, shock index > 1.2, presence of hepatocellular carcinoma (HCC), red color (RC) signs, endoscopic active bleeding, CTP score, white blood cell count (WBC), neutrophil percentage (NE%), hemoglobin (HGB), prothrombin time (PT), activated partial thromboplastin time (APTT), total bilirubin (TBIL), and sodium (Na). These features were subsequently utilized in a multivariate logistic regression analysis.

### 3.3 Development of a predictive model for endoscopic hemostasis treatment failure

**Figure 3:**
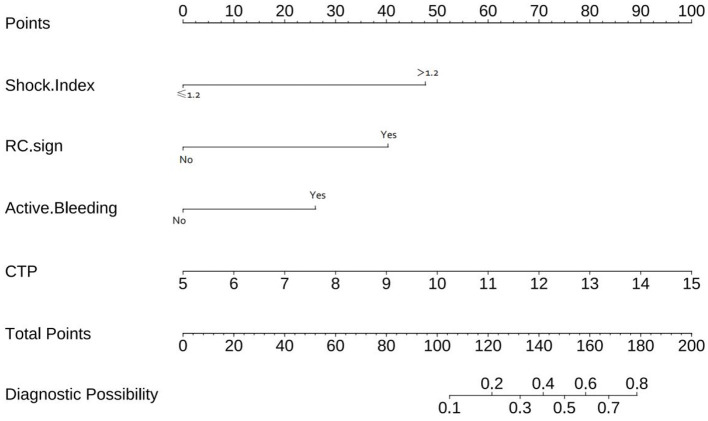
The nomogram for predicting the endoscopic hemostatic treatment efficacy in patients with cirrhotic EGVB assigns a score between 1 and 100 to each variable. These individual scores are subsequently aggregated to produce a total score. This cumulative score is then aligned on the total score axis of the nomogram, facilitating the estimation of the probability of rebleeding risk in patients with liver cirrhosis and EGV.

The multivariate logistic regression analysis results (as presented in Table 2) facilitated the derivation of a risk score aimed at predicting the failure of endoscopic treatment in cases of cirrhotic EGVB. The logistic regression equation is expressed as: Logit (*P*) = −3.548 + 1.695 ^*^ *X*1 + 2.303 ^*^ *X*2 + 1.785 ^*^ *X*3 + 0.46 ^*^ *X*4. In this equation, *X*1 represents a SI >1.2, *X*2 denotes the presence of RC Sign, *X*3 indicates Endoscopic Active Bleeding, and *X*4 corresponds to the CTP Score. Additionally, a nomogram was developed to estimate the probability of endoscopic hemostasis treatment failure in cirrhotic EGVB, which is mathematically defined as *P* = 1/[1 + e^Λ^ (−3.548 + 1.695 ^*^ *X*1 + 2.303 ^*^ *X*2 + 1.785 ^*^ *X*3 + 0.46 ^*^ *X*4)] (refer to Figure 3).

**Table 2:** Analysis of influencing factors of emergency endoscopic hemostasis treatment in EGVB patients based on multivariate logistic regression.

**Characteristics**	**B**	**SE**	**OR**	**CI**	***Z***	***P***
(Intercept)	−3.548	2.39409	0.029	0.028 (0.000–2.796)	−1.482	0.138
Shock index > 1.2	1.695	0.60035	5.447	5.447 (1.727–18.76)	2.824	0.005^**^
RC sign	2.303	0.85436	10.005	10.00 (2.307–74.70)	2.696	0.007^**^
Active bleeding	1.785	0.66562	5.962	5.961 (1.633–23.26)	2.682	0.007^**^
CTP	0.46	0.12877	1.584	1.584 (1.242–2.071)	3.573	< 0.001^***^

^**^P < 0.01, ^***^P < 0.001.

### 3.4 Validation of the predictive accuracy and clinical utility of the nomogram model

**Figure 4:**
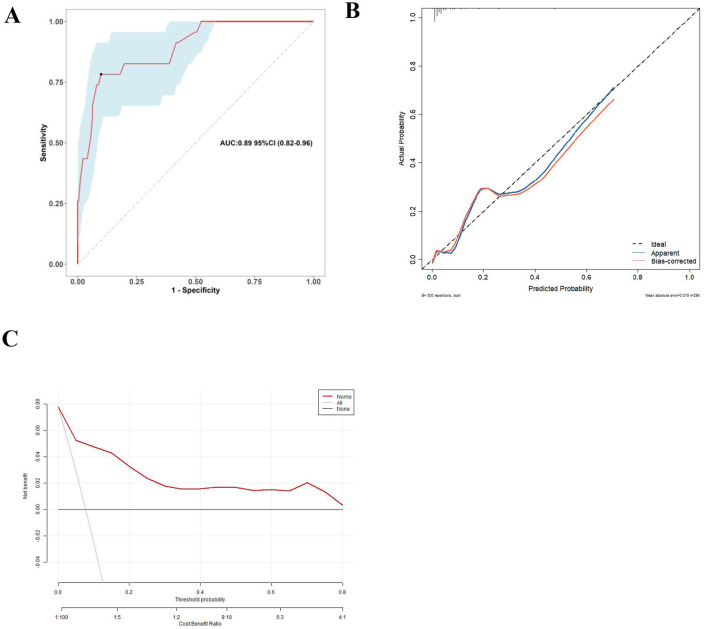
**(A)** The ROC curve of the nomogram, evaluated through 500 bootstrap iterations. **(B)** The calibration curve for rebleeding predictions generated by the nomogram. The proximity of the Apparent line and the Bias-corrected line to the ideal line indicates enhanced predictive accuracy of the nomogram. **(C)** DCA for the predictive model, where the red line represents the predictive model, the gray line represents the net benefit of the “Treat All” strategy of performing endoscopic treatment on all patients, and the black line represents the net benefit of the “Treat None” strategy of not treating any patients.

The predictive accuracy of the nomogram model was evaluated and validated through internal bootstrapping. Employing 500 bootstrap resamples to mitigate overfitting, the AUC for the cohort was determined to be 0.890, with a 95% confidence interval ranging from 0.820 to 0.960. The calibration curve indicated a strong concordance between predicted and observed probabilities, as evidenced by the Hosmer–Lemeshow test (χ^2^ = 10.021, *P* = 0.263). Furthermore, DCA conducted after 500 bootstrap samples demonstrated that the nomogram model offered a positive net benefit over “treat all” or “treat none” strategies across a broad spectrum of threshold probabilities (refer to Figure 4).

### 3.5 Risk stratification and clinical intervention thresholds utilizing the nomogram model

Patients were categorized into three distinct groups according to the quartile distribution of total scores as predicted by the nomogram model: the low-risk group (total score ≤ 50.30, representing the 25th percentile) comprised 86 cases; the medium-risk group (total score between 50.30 and 94.27, corresponding to the 25th to 75th percentile range) included 136 cases; and the high-risk group (total score > 94.27, exceeding the 75th percentile) consisted of 74 cases. A significant upward trend in treatment failure rates was observed across these groups (low-risk: 0.00%, medium-risk: 5.70%, high-risk: 19.20%, *P* < 0.001). Based on this stratification, the proposed clinical intervention strategies are as follows: patients classified within the high-risk group should receive enhanced monitoring of vital signs post-endoscopic treatment, with a recommendation for transfer to ICU care. In the event of recurrent active bleeding, immediate secondary endoscopic intervention, balloon tamponade using dual-lumen tubes, or planned procedures such as TIPS should be promptly administered.

### 3.6 Predictive performance and clinical value of CTP, MELD, rockall scores, and the nomogram model

**Figure 5:**
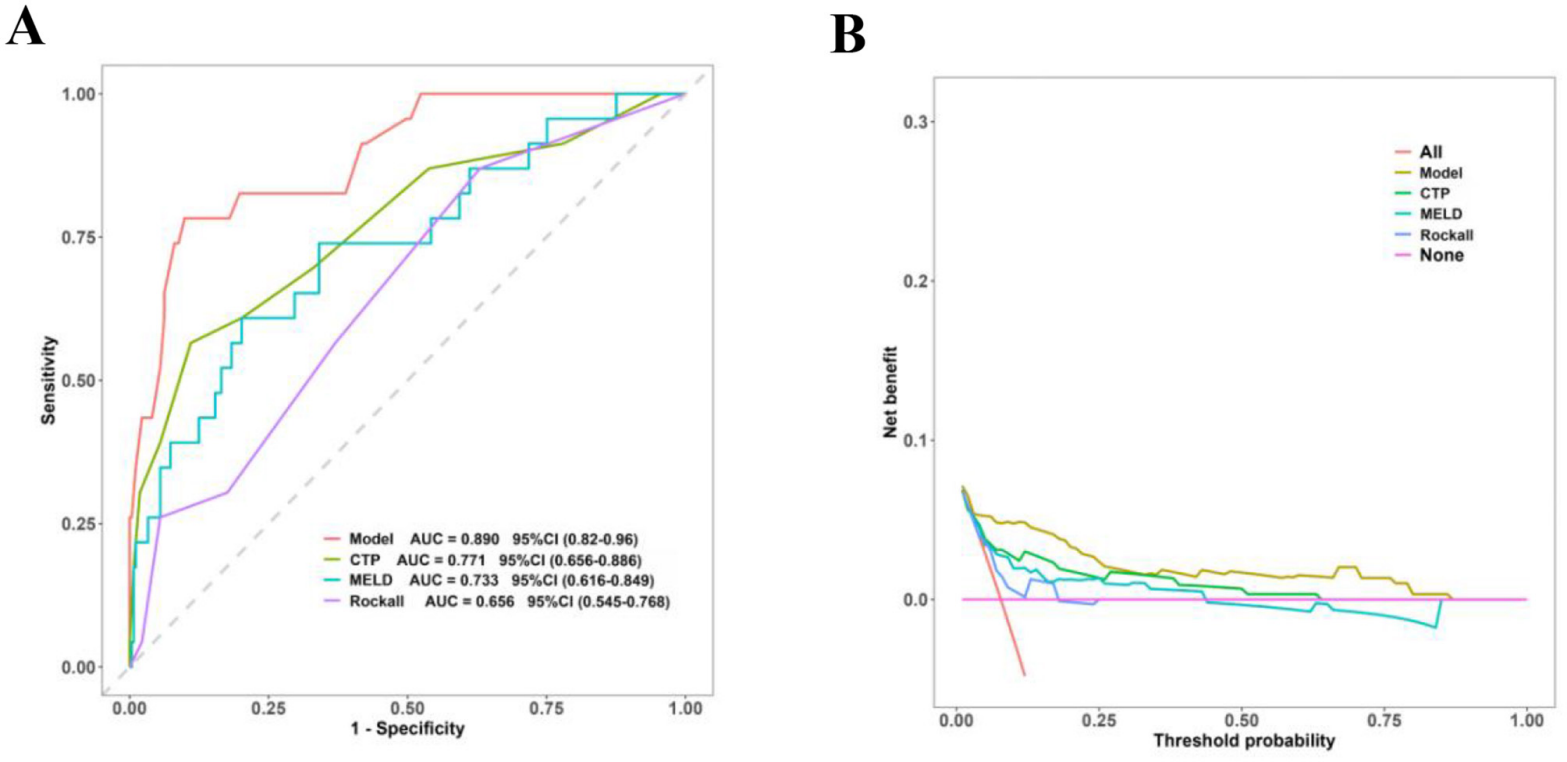
**(A)** ROC curves comparing the performance of the nomogram with other established clinical scoring systems. **(B)** DCA evaluating the clinical utility of the nomogram in relation to other established clinical scoring systems.

The analysis of the ROC curve for the nomogram model revealed an AUC of 0.890 (95% CI: 0.820–0.960), which was significantly superior to that of the CTP score (AUC = 0.771, 95% CI: 0.656–0.886; DeLong test, *P* < 0.001), MELD score (AUC = 0.733, 95% CI: 0.616–0.849; *P* < 0.001), and Rockall score (AUC = 0.656, 95% CI: 0.545–0.768; *P* < 0.001) (Figure 5). Additionally, we employed the Integrated Discrimination Improvement (IDI) and Net Reclassification Improvement (NRI) metrics to evaluate the discriminative capabilities of the nomogram model in comparison to other clinical scoring systems. Our findings suggest that the nomogram model exhibits enhanced accuracy in predicting the failure of endoscopic hemostasis in patients with cirrhotic EGVB, underscoring its potential as a valuable clinical tool (Table 3). The DCA for CTP, MELD, Rockall, and the nomogram model is illustrated in Figure 5. In the context of predicting treatment failure in cirrhotic EGVB, the nomogram model consistently offered greater net benefits across a broad spectrum of threshold probabilities when compared to the CTP, MELD, and Rockall scores.

**Table 3:** Comparison of nomogram model and clinical scoring systems: IDI and NRI analyses.

**Variable**	**NRI (95%CI)**	***P*-value**	**IDI (95%CI)**	***P*-value**
Model CTP	1.198 (0.873–1.523)	< 0.001^***^	0.165 (0.092–0.239)	< 0.001^***^
Model MELD	1.199 (0.850–1.548)	< 0.001^***^	0.228 (0.134–0.322)	< 0.001^***^
Model Rockall	1.170 (0.819–1.519)	< 0.001^***^	0.291 (0.172–0.410)	< 0.001^***^

^***^P < 0.001.

## 4 Discussion

In recent years, advancements in endoscopic treatment techniques and the accumulation of clinical experience have facilitated the widespread adoption of a multidisciplinary collaborative model for the emergency endoscopic management of cirrhotic EGVB throughout the disease's progression. In cases of refractory hemorrhagic shock, the collaboration of a multidisciplinary team allows for the prompt execution of emergency endoscopy and treatment, thereby enhancing patient survival prospects (11). Nevertheless, despite ongoing technological progress, 10% to 20% of patients still experience hemostasis failure during emergency endoscopic procedures, which significantly elevates the risk of mortality for those unable to achieve hemostasis (12). In this study, the failure rate of emergency endoscopic hemostasis for cirrhotic EGVB at our institution was 7.80%, aligning with findings reported in some literature (13, 14). Consequently, in light of the aforementioned circumstances, it is imperative to identify high-risk populations, implement rescue measures at the earliest opportunity, and develop preventive strategies and emergency plans proactively. Simultaneously, for patients whose bleeding remains uncontrolled or who experience re-bleeding, timely salvage treatment should be administered to minimize the risk of hemostasis failure.

Numerous factors influence the outcomes of endoscopic treatment in patients with cirrhosis and EGVB, including portal vein pressure (15), the presence of ascites (16), timing of intervention (13), and liver function status (17). This study examined 30 variables that may impact the endoscopic treatment outcomes of EGVB in cirrhotic patients, identified independent predictors, and developed a nomogram model. This model emphasizes the SI, endoscopic red color signs, active bleeding, and the CTP score as key predictive factors. Notably, the Shock Index, which is calculated as the ratio of heart rate to systolic blood pressure, is widely utilized in the assessment of various acute medical conditions (9). In the context of hemodynamic instability, the shock state indicates that tissue hypoperfusion may impact treatment outcomes through several mechanisms. Firstly, in patients experiencing hemorrhagic shock, significant bleeding can obscure the endoscopic field of vision, thereby increasing the technical complexity of the procedure and elevating the risk of treatment failure (18, 19). Secondly, inadequate perfusion of the microcirculation can result in tissue hypoxia and the accumulation of metabolic byproducts, which may impair the synthesis and function of coagulation factors, leading to coagulopathy (20). Lastly, tissue hypoperfusion can exacerbate the limited functional reserve of the liver, potentially resulting in acute liver injury and adversely affecting patient prognosis (21). A retrospective study (22) analyzed electronic medical records of patients hospitalized with Upper Gastrointestinal Bleeding (UGIB), gathering data on patient demographics, clinical presentations, comorbidities, endoscopic findings, and outcomes. This study confirmed the significant utility of the SI in identifying UGIB patients at risk for adverse outcomes. Sun et al. (23) demonstrated that the Shock Index is straightforward to monitor, aids in dynamic assessment, and can be utilized for risk stratification in portal hypertension. The importance of endoscopic red color signs and active bleeding highlights the fundamental value of dynamic endoscopic features; red color signs suggest increased vulnerability of the venous wall structure and are positively associated with the risk of rebleeding (24), whereas active bleeding observed endoscopically indicates that uncontrolled hemorrhage may compromise the accuracy of hemostatic procedures (3). Research conducted by Orloff et al. (25) indicated that the CTP score is highly valuable in predicting the prognosis of endoscopic treatment for EGVB in cirrhosis. The inclusion of the CTP score in this study further corroborates the pivotal role of liver dysfunction in influencing treatment response. Its interaction with the shock state may elucidate the model's capacity to capture the systemic-local interactive effects.

This study is pioneering in three principal dimensions: Firstly, To the best of our knowledge, this study is among the first to systematically investigate and validate the combined utility of “immediate endoscopic features during acute variceal bleeding” and “systemic status indicators” in predicting the failure of endoscopic treatment. Secondly, in comparison to the hepatic venous pressure gradient (HVPG) model endorsed by the Baveno VII consensus (2), this model demonstrates comparable predictive performance utilizing non-invasive indicators, thereby enhancing its accessibility for primary care environments. Lastly, the application of LASSO regression facilitated the optimization of variable selection, effectively mitigating overfitting issues associated with multicollinearity (26). This study, however, is not without its limitations. Firstly, as a single-center retrospective investigation, the sample size was relatively limited (*n* = 296), with only 23 cases categorized within the treatment failure group. Although internal validation was conducted using bootstrap resampling (500 iterations), there remains a necessity for external validation through multicenter, prospective, large-scale cohorts in subsequent research. Secondly, imaging parameters, such as portal vein diameter, were excluded from the model due to insufficient data integrity. Future studies should incorporate data from CT portal venography or ultrasound elastography to enhance and refine predictive efficacy. Furthermore, the relatively short observation period for patients restricted the comprehensive assessment of long-term prognosis. Future research directions include integrating CT portal vein imaging or ultrasound elastography to acquire vascular parameters, investigating the incremental value of novel biomarkers such as serum microRNAs, and developing AI-based automatic endoscopic image analysis modules for quantitative assessment of red color signs. Ultimately, the goal is to construct a clinical decision support system (CDSS) by embedding the prediction threshold.

## 5 Conclusion

In conclusion, this study successfully developed and validated a nomogram model incorporating the SI, RC sign, endoscopic active bleeding, and CTP score to predict adverse outcomes following endoscopic hemostatic treatment in patients with cirrhotic EGVB. The model exhibited excellent discrimination, with an AUC of 0.890, and demonstrated superior calibration compared to traditional scoring systems such as CTP, MELD, and Rockall scores. Furthermore, the inclusion of a visual interface facilitates rapid risk stratification. The clinical utility of this model lies in its capacity to effectively identify high-risk patients at an early stage, thereby enabling the timely implementation of rescue measures to address potential complications.

## Data Availability

The original contributions presented in the study are included in the article/supplementary material, further inquiries can be directed to the corresponding authors.
